# Exploring chronic pain: demographic and social determinants of health insights in Maine

**DOI:** 10.3389/fpain.2026.1763519

**Published:** 2026-04-13

**Authors:** Penhleakhena Ou, Elizabeth N. Bean, Ling Cao

**Affiliations:** 1 University of New England College of Osteopathic Medicine, Biddeford, ME, United States; 2 Department of Biomedical Sciences, University of New England College of Osteopathic Medicine, Biddeford, ME, United States; 3 Center for Pain Research, University of New England, Biddeford, ME, United States

**Keywords:** chronic pain, disparities, pain registry, PROPr, REDCap, social determinants of health

## Abstract

**Introduction:**

Chronic pain is a serious public health problem in Maine. We initiated a pain registry for the adult population across Maine to characterize the population with persistent or recurrent pain, with a special focus on the relationships between social determinants of health (SDOH) and chronic pain outcomes (pain level and co-morbidities) and management, therefore, to identify targeted public health interventions to improve pain care in Maine.

**Methods:**

The registry uses the REDCap data collection platform to administer an anonymous survey, which includes questions regarding pain characteristics, pain management, and demographic and SDOH factors. The survey was distributed through both hard- and electronic copies of a flyer.

**Results:**

The current study reports the initial cross-sectional descriptive analysis of the first 109 respondents with the focus on the relationships between selected socio-demographic factors and pain outcome measures. Compared to Maine's census data, the current participant pool is overrepresented in biological females, individuals 65 and older, minority racial/ethnic groups, and education attainment, but underrepresented in veterans. The majority of participants experienced pain for more than a year and described their pain as moderate to severe. Low back was the most frequently reported pain location, and trauma/injury was the most frequently identified cause. Fatigue, pain interference, reduced physical function, and sleep disturbance were major co-morbidities. Higher SDOH risk was significantly associated with worse pain-related outcomes. Being younger and having lower education levels were associated with increased anxiety and depression but reduced cognitive function. Lower education level was also associated with greater fatigue and pain score. The White-only race was associated with lower levels of anxiety, depression and sleep disturbance. Within the current participant pool, biological sex, veteran status and rural living did not significantly affect any pain outcomes.

**Discussion:**

In summary, additional recruitment efforts to reach all socio-demographic groups are warranted and further data analysis could inform research in pain and pain treatment as well as public health strategies for pain prevention in Maine.

## 1 Introduction

Chronic pain is a significant and costly public health concern in the United States (US). The annual cost of pain in the US ranges from $560 to $635 billion and is attributed to both health care costs and costs due to loss of productivity, with the amount exceeding the annual costs of heart disease, cancer, and diabetes, respectively (1). According to the Centers for Disease Control and Prevention (CDC), approximately 24% of US adults are affected (2). Those living with chronic pain can have reduced quality of life, frequent healthcare utilization, and comorbidities such as depression and anxiety (3). The experience of chronic pain is complex and influenced by multiple factors including but not limited to demographics, lifestyle behaviors, physical activity, and mental health (4). Collectively, these components are encompassed within the framework of social determinants of health (SDOH), which are increasingly recognized as critical influences on the development and management of chronic pain (5–7).

National surveillance data, such as those reported by the CDC in 2024, indicate that the prevalence of chronic pain is disproportionately higher among certain subpopulations, including older adults, females, and those living in nonmetropolitan areas (2). Geographic variation plays a role in the burden of chronic pain, with some states experiencing higher prevalence rates than others. For example, a study by Malon et al. (2018) examined chronic pain in Maine using insurance claims data (2006–2011) and identified 29.5% of Mainers have chronic pain (8), which is similar to a national-wide web-based survey study (30.7%) conducted in 2008–2009 for US adults but greater than the national average published by the CDC (20.4%) obtained through the 2016 US adult National Health Interview Survey (NHIS) data (9, 10). The prevalence of chronic pain in Maine increased with age specifically for those over 65 and it was found that the burden of chronic pain is significantly higher in females, patterns which are consistent with epidemiology studies conducted over the past decades (2, 9–11).

Maine presents a particularly unique landscape for chronic pain research. According to the 2024 U.S. Census data, Maine has the highest proportion of residents aged 65 years and older, a demographic that has a high prevalence of chronic pain. Despite this, literature within the state remains limited. Current knowledge of chronic pain prevalence in Maine is limited to the sole study from Malon et al. (2018) (8), which used the Maine all payers insurance claims database from more than a decade ago. While understanding the distribution of who experiences chronic pain in the state is useful, this dataset does not provide insight into how SDOH such as race, veteran status, rurality, and mental health intersect to influence chronic pain and its associated burden.

The absence of studies with robust information on the impact of SDOH on chronic pain and its management and the lack of qualitative data regarding chronic pain in Maine warrants a SDOH-focused data collection and analysis. Such research can inform more equitable and effective public health strategies, healthcare policies, and clinical interventions tailored to at-risk populations. The objectives of our research team are to identify a broader range of SDOH factors that may affect chronic pain in Maine and describe how specific factors can influence individuals’ pain experiences. In this study, we took advantage of our newly established SDOH-focused pain registry in Maine and conducted the analysis with the “first-look” of the data collected from the registry. It is our hope that our findings can contribute to a better understanding of chronic pain within the state and hopefully offer insights applicable to similarly structured rural and aging populations.

## 2 Materials and methods

### 2.1 Overview

Through a pilot grant from the Northern New England Clinical & Translational Research Network (NNE-CTR), in collaboration with MaineHealth, we initiated a longitudinal SDOH-focused pain registry (PainRegistryforME) designed to investigate the relationships between chronic pain and SDOH among adults residing in Maine. The registry was launched in the summer of 2023 and continues to collect data with the goal of generating a robust dataset that captures both the clinical and contextual aspects of chronic pain across communities in the state. This PainRegistryforME study was reviewed and approved by the MaineHealth Institutional Review Board (IRB) (IRBnet ID:1970910).

### 2.2 Participants and recruitment

Individuals living in the state of Maine who are ≥ 18 years old with any type of persistent or recurrent pain are eligible to participate. Persistent or recurrent pain is defined as pain experienced most days or every day. To ensure accessibility and confidentiality, the study utilized an anonymous, self-administered online survey hosted on REDCap (Research Electronic Data Capture), a secure, HIPAA-compliant survey instrument. Study data were collected and managed using REDCap electronic data capture tools hosted at University of New England (12, 13). REDCap is a secure, web-based software platform designed to support data capture for research studies by providing 1) an intuitive interface for validated data capture; 2) audit trails for tracking data manipulation and export procedures; 3) automated export procedures for seamless data downloads to common statistical packages; and 4) procedures for data integration and interoperability with external sources.

The survey link and Quick Response (QR) code was distributed virtually and physically through institutional networks, collaborating clinics, and chain grocery stores via the study flyer. To target areas with high prevalence of patients with chronic pain, clinical collaborators including specialists in pain management and opioid use disorder assisted with outreach. To reach potential participants throughout the state, clinical collaborators within the University of New England clinical training partners and Northern Light Eastern Maine Medical Center assisted in flyer distribution. Specifically, Northern Light Eastern Maine Medical Center has connections with Maine rural residents. Additional dissemination occurred through seminars, email listservs, social media postings, and professional association events. This recruitment strategy was designed to capture a wider geographic and socioeconomic diversity among respondents. In this study, we used the results from the “First Look” interim analysis of the data that included data collected up to February 2024. A total of 109 out of 112 participants had entered responses and their data were eligible for analysis in this study. The remaining 3 participants had empty entries for the entire survey and therefore could not be used.

### 2.3 Survey design and measures

The PainRegistryforME survey has a total of 75 questions organized into four major sections: (1) Demographics, (2) Pain Characteristics, (3) Pain Management, and (4) Social Determinants of Health (SDOH). SDOH factors were assessed using the Upstream Risks Screening Tool by HealthBegins, one of the first developed comprehensive SDOH screening tools (14, 15). Due to the relatively small participant pool, selected key demographic variables related to SDOH were used in this initial data analysis. These variables included age, biological sex, gender, veteran status, race, and county of residence. Education level was assessed based on the highest degree earned and higher level of schooling completed. Other SDOH variables related to housing, employment, and healthcare access are also collected in the full registry and will be explored in future analyses.

Pain characteristics and related health outcomes were assessed using the NIH Patient-Reported Outcomes Measurement Information System (PROMIS®) 29 + 2 Profile v2.1 (PROPr), a validated instrument widely used to evaluate health-related quality of life in individuals with chronic conditions and available through RedCap (16). This tool includes 31 questions divided into eight health domains: anxiety, depression, fatigue, pain interference, physical function, sleep disturbance, ability to participate in social roles and activities, and cognitive function. Participants respond to each item on a 5-point Likert scale and scores are then summed per domain to obtain domain scores. Additionally, a single-item numeric rating scale was used to assess pain intensity in the past 7 days, with values ranging from 0, indicating no pain, to 10, indicating the worst possible pain. Furthermore, the calculated single preference-based PROPr score obtained using the scores for 7 PROMIS domains (depression, fatigue, pain interference, physical function, sleep disturbance, ability to participate in social roles and activities, and cognitive function) for each participant could be used to represent the individual health state related to chronic pain with 1 indicating the optimal health and 0 indicating the worst outcome such as death (17).

### 2.4 Data analysis

Descriptive statistics were performed using Microsoft® Excel to characterize the sample and provide a profile of demographic and SDOH distributions. For further statistical analysis, individual one-way ANOVA (ANOVA) was conducted via IBM SPSS Statistics (Version 29, Armonk, NY) to assess the associations between categorical variables (demographics and SDOH factors) and pain outcome variables measured across 8 individual PROMIS domains. For each ANONA test, pre-tests were conducted to confirm the statistical assumptions for running each one-way ANOVA test. Given this is the first look of the data collected in the registry, we have no clear evidence regarding how individual factors we examined were interacting with each other or any of them were confounders. Therefore, there is no solid evidence for adjusting *p* values when conducting each one-way ANOVA individually. *p* < 0.05 was considered to be statistically significant. Data were graphed using SigmaPlot 14.5 (Systat Software Inc., San Jose, CA). When comparisons to Maine's census data were made, the 2024 Maine Census data were used unless specified otherwise.

## 3 Results

Out of the 112 respondents, data from 3 respondents were excluded from the data analysis due to missing data in all fields, leaving 109 subjects eligible for analysis. Therefore, all percentages were calculated based on the total sample size as 109. For any analysis, if a participant provided no response (blank) to a particular question, the participant was not included in the sub-category analysis involving this question.

### 3.1 Socio-demographic profile of participants

Demographic and selected SDOH characteristics are summarized in Table 1.

**Table 1:** Demographic and SDOH characteristics of respondents (total *N* = 109).

REDCap Survey Questions		*N*	Frequency (%)
What is your age?	18–24 years	7	6.4
	25–29 years	12	11.0
	30–34 years	7	6.4
	35–39 years	9	8.3
	40–45 years	7	6.4
	45–49 years	6	5.5
	50–54 years	4	3.7
	55–59 years	12	11.0
	60–64 years	10	9.2
	65–69 years	8	7.3
	70–74 years	11	10.1
	75–79 years	8	7.3
	80 years and above	8	7.3
What is your biological sex?	Female	76	69.7
	Male	30	27.5
	Prefer not to answer	1	0.9
	No response [blank]	2	1.8
What is your gender (what you identify yourself as)?	Female	73	67.0
	Male	29	26.6
	Transgender	3	2.8
	Non-binary	1	0.9
	Prefer not to answer	1	0.9
	No response [blank]	2	1.8
Are you Hispanic or Latino?	Yes	9	8.3
	No	94	86.2
	No response [blank]	6	5.5
Which race(s) are you?	American Indian/Alaskan Native only	2	1.8
	Asian only	3	2.8
	Black/African American only	6	5.5
	Native Hawaiian only	0	0.0
	Pacific Islander only	1	0.9
	White only	92	84.4
	Mixed	5	4.6
Which county in Maine do you reside in?	Androscoggin	6	5.5
	Aroostock	3	2.8
	Cumberland	40	36.7
	Franklin	3	2.8
	Hancock	5	4.6
	Kennebec	4	3.7
	Knox	1	0.9
	Lincoln	3	2.8
	Oxford	2	1.8
	Penobscot	3	2.8
	Piscataquis	1	0.9
	Sagadahoc	0	0.0
	Somerset	1	0.9
	Waldo	2	1.8
	Washington	0	0.0
	York	35	32.1
Are you a US military veteran?	Yes	6	5.5
	No	100	91.7
	No response [blank]	3	2.8
What is the highest level of school you have completed?	Elementary	0	0.0
	High	15	13.8
	College	53	48.6
	Graduate/Professional	40	36.7
	Prefer not to answer	1	0.9
What is the highest degree you earned?	High School	13	11.9
	GED	0	0.0
	Vocational (Post high school or GED)	3	2.8
	Associate's degree	9	8.3
	Bachelor's degree	49	45.0
	Master's degree	23	21.1
	Doctorate	11	10.1
	Prefer not to answer	1	0.9

In this initial cohort, we had participants in every age group with the age groups 25–29 years of age and 55–59 years of age having the greatest numbers of participants. Each of these groups had 12 participants (12/109, 11.0%). The participant pool was overrepresented by individuals 65 and above (35/109, 32.1%) compared to the Maine's census data (22.5%).

Both questions regarding biological sex and gender were asked in the survey. Our respondents predominantly reported as female sex (76/109, 69.7%) and female gender (73/10, 67.0%). Therefore, our participant pool is overrepresented by females compared to the Maine state census data (50.7%).

Our respondent pool was more diverse than Maine's general population reported in the census data. Among the 109 participants, 92 respondents reported to be White only (84.4% vs. 93.9% in Maine's census), 6 to be Black/African American only (5.5% vs. 2.0% in Maine's census), and 9 participants had Hispanic origin (8.3% vs. 2.0% in Maine's census).

Our results showed 5.5% (6/109) of respondents being veterans, which is underrepresented per the Maine census data that reported about 7.2% of the population being veterans.

In regards to county of residence, 77.1% (84/109) of respondents lived in urban counties in Maine which includes York, Cumberland, Androscoggin, Sagadahoc and Penobscot counties (18). The majority lived in Cumberland and York counties (75/109, 68.8%), reflecting the fact that these are the two most populated counties in Maine (32.1% and 15.7% of the Maine's population, respectively).

Two different sets of questions in the registry pertained to education level. The first asked respondents what their highest educational degree earned is and the other question asked the highest level of school completed. Our respondents were overrepresented by a highly educated population compared to Maine's general population, with 3 in 4 participants possess a Bachelor's degree or higher (83/109, 76.1%, vs. 37.1% in Maine's census data), and 4 in 5 participants completed some college or graduate/professional school [93/109, 85.3% vs. 66.6% in the MaineSpark-2023 report (19)].

### 3.2 Pain characteristics of participants

**Figure 1:**
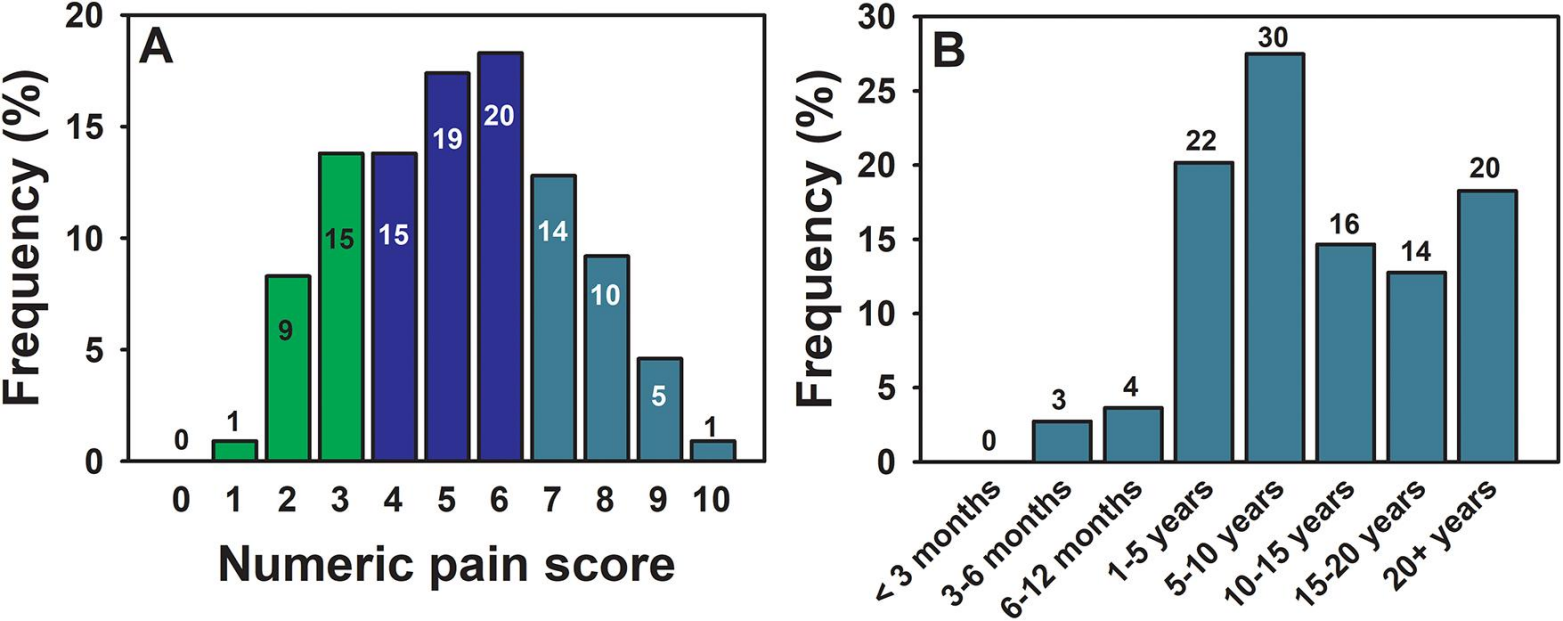
The numeric pain score and the reported pain-experiencing times of the current participant pool. Data are shown for the 109 participants. In **(A)**, the frequency of each numeric score (0-10) is presented and numbers of participants with each score are shown within each colored bar. Score 0 = no pain, 1-3 = minor pain, 4-6 = moderate pain, and 7-10 = severe pain. In **(B)**, the frequency of each pain-experiencing duration reported by participants are shown. The numbers of participants with each duration are listed on top of each bar.

Most respondents experienced moderate to severe pain according to the 0–10 numeric pain score (survey question: *In the past 7 days: How would you rate your pain on average? Note that: 0* *=* *No pain and 10* *=* *The worst possible pain*) (5.25 ± 2.01) (Figure 1A). All respondents met the chronic pain diagnosis as all subjects reported having pain for at least 3 months (20) when responding to the question “*How long have you been experiencing your pain?*”. Most respondents had pain for 1 year or longer and approximately 1 in 5 participants had pain for 20 years or longer (Figure 1B), with the longest stated as 52 years.

Other pain characteristics are summarized in Table 2. Nearly half experienced pain in the low back area (52/109, 47.7%) followed by pain experienced in shoulder(s) (31/109, 28.4%) and neck area (30/109, 27.5%) (Table 2). Other reported locations that were not in the provided list mostly included extremities (7/109, 6.4%) and abdomen (3/109, 2.8%). More than half of the participants (including 7 individuals who selected “whole body”) selected more than one location in their responses (61/109, 56.0%).

**Table 2:** Selected pain characteristics of respondents (total *N* = 109).

REDCap Survey Questions		*N*	Frequency (%)
Where is your pain located? (Check what best describes your pain; check all that apply)	Whole body	19	17.4
	Most of the upper body	5	4.6
	Most of the lower body	9	8.3
	Most of the left side (upper and lower body)	7	6.4
	Most of the right side (upper and lower body)	2	1.8
	Head	12	11.0
	Oral facial	10	9.2
	Neck	30	27.5
	Upper Back	19	17.4
	Lower Back	52	47.7
	Shoulders	31	28.44
	Primarily joint(s) (large and small, except shoulders)	23	21.1
	Primarily limb(s) (upper or lower)	20	18.4
	Other	12	11.0
What do you think was the original cause of your pain (Please choose one only)?	Trauma/Injury	39	35. 8
	Surgery procedure(s)	9	8.3
	Chronic non-cancer illness (such as arthritis	30	27.5
	Cancer	1	0.9
	Other	26	23.9
	Prefer not to answer	4	3.7
	No response [blank]	5	4.6

As for precipitating events, more than a third of the participants reported the pain was due to trauma or injury (39/109, 35.8%), which is followed by chronic non-cancer illness (30/109, 27.5%) (Table 2). Other causes written in by participants mainly included excessive usage, genetic, emotional status, and combination of multiple factors.

Another important set of pain characteristic outcome measures were obtained from the PROPr instrument assessing pain-related comorbidities. The sum score for each of the 8 domains anxiety, depression, fatigue, pain interference, physical function, sleep disturbance, ability to participate in social roles and activities, and cognitive function, as well as the calculated preference-based PROPr scores are summarized in Table 3.

**Table 3:** Summary of pain comorbidities measured by the PROPr instrument^a^.

Domain name	Mean ± SD
Increased morbidity with higher scores	
Anxiety (up to 20)	8.9 ± 3.9
Depression (up to 20)	8.4 ± 4.2
Pain interference (up to 20)	11.4 ± 4.1
Fatigue (up to 20)	12.1 ± 4.2
Sleep disturbance (up to 20)	11.2 ± 1.9
Increased morbidity with lower scores	
Physical function (up to 20)	8.7 ± 3.6
Ability to participate in social roles and activities (up to 20)	11.3 ± 3.7
Cognitive function (up to 10)	7.8 ± 1.9
PROPr score (range 0–1, with 0 = worst outcome and 1 = optimal health)	0.29 ± 0.17

^a^ There were no missing responses for the PROPr instrument, however, one subject's responses did not yield an overall PROPr score via the online calculation portal.

### 3.3 Socio-demographic impact on pain-related outcomes

**Figure 2:**
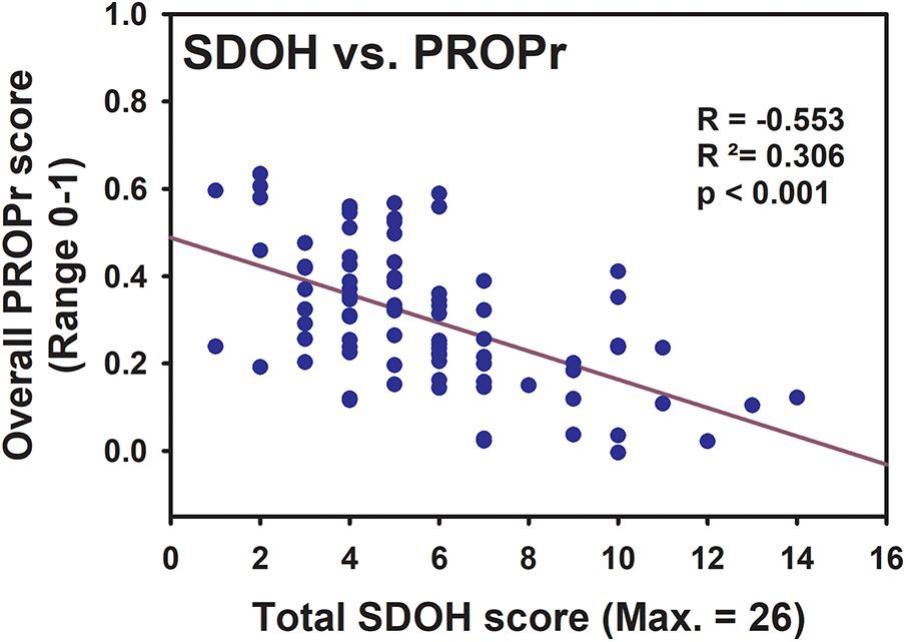
Relationship between the total SDOH score and the overall PROPr score. Data from 85 participants were available for both scores and presented here. The regression line, R, R^2^, and *p* value from the Pearson Correlation are shown.

To assess the relationship between SDOH and pain outcomes, we first calculated the total SDOH scores (higher score = higher risk) for each participant from the Upstream Risks Screening Tool by HealthBegins according to its guidelines, and examined its association with the calculated overall preference-based PROPr score. Although we were able to obtain the PROPr scores for all participants except one individual, the total SDOH score could only be generated for participants who completed all fields of the SDOH portion (near 30 questions) of the survey (total of 86) while excluding those who marked the “prefer not to answer” option for any question or left any field blank. Therefore, 85 out of 109 individuals were included in the association test between the total SDOH score and the PROPr score. Note that there did not appear to be any common characteristics within the group that did not have a total SDOH score. Therefore, we do not believe this has resulted in any systemic error. With the available scores from 85 participants, it was found that SDOH score was significantly negatively correlated with the PROPr score (Figure 2, Pearson correlation, correlation coefficient = −0.553, *p* = 4.11 × 10^−8^).

Next, we examined the relationships between selected socio-demographic factors and each of the 8 pain outcome domains used by the PROPr instrument: anxiety, depression, fatigue, pain interference, physical function, sleep disturbance, ability to participate in social roles and activities, and cognitive function, plus the numeric pain score. Note that the two questions asked in cognitive function domain had to do with the subject's ability to concentrate and remember common tasks.

**Figure 3:**
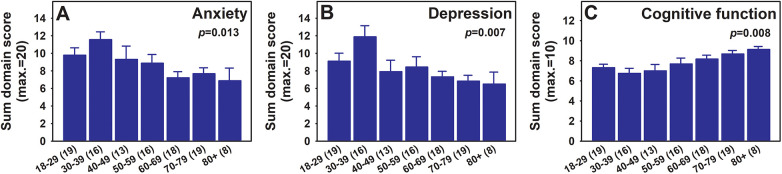
PROPr anxiety **(A)**, depression **(B)**, and cognitive function **(C)** scores across age groups. The mean sum scores for each indicated outcome domain are presented across all age groups (mean ± SEM). One-way ANOVA *p* values are shown in respective graphs. Numbers of participants within each group are listed with respective group names in parenthesis on the X-axis. Note that higher scores for anxiety and depression correlate to worse outcomes, while higher score for cognitive function correlates to better outcomes.

Age was collected in 5-year interval in the survey but due to the limited sample size within each 5-year interval, age was collapsed into 10-year ranges for analysis. Although the prevalence of chronic pain increases with age, in our current participant pool, being younger was associated with worse pain-related outcomes including anxiety (Figure 3A, one-way ANOVA, *p* = 0.013), depression (Figure 3B, one-way ANOVA, *p* = 0.007), and cognitive function (Figure 3C, one-way ANOVA, *p* = 0.008).

**Figure 4:**
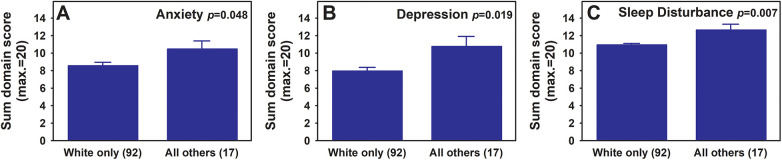
PROPr anxiety **(A)**, depression **(B)**, and sleep disturbances **(C)** scores for white only race vs. all others combined. The mean sum scores for each indicated outcome domain are presented for both race groups (mean ± SEM). One-way ANOVA *p* values are shown in respective graphs. Numbers of participants within each group are listed with respective group names in parenthesis on the X-axis. Note that for all three domains, higher scores correlate to worse outcomes.

Regarding race and ethnicity, due to the small sample sizes for non-White or Hispanic populations in our current participants, comparisons were made between White only and all others combined. Significant differences between White only and all others were identified in the following 3 PROPr outcome domains: anxiety (Figure 4A, one-way ANOVA, *p* = 0.048), depression (Figure 4B, one-way ANOVA, *p* = 0.019), and sleep disturbance (Figure 4C, one-way ANOVA, *p* = 0.007), with those who identified as White only having better outcomes in all three domains.

**Figure 5:**
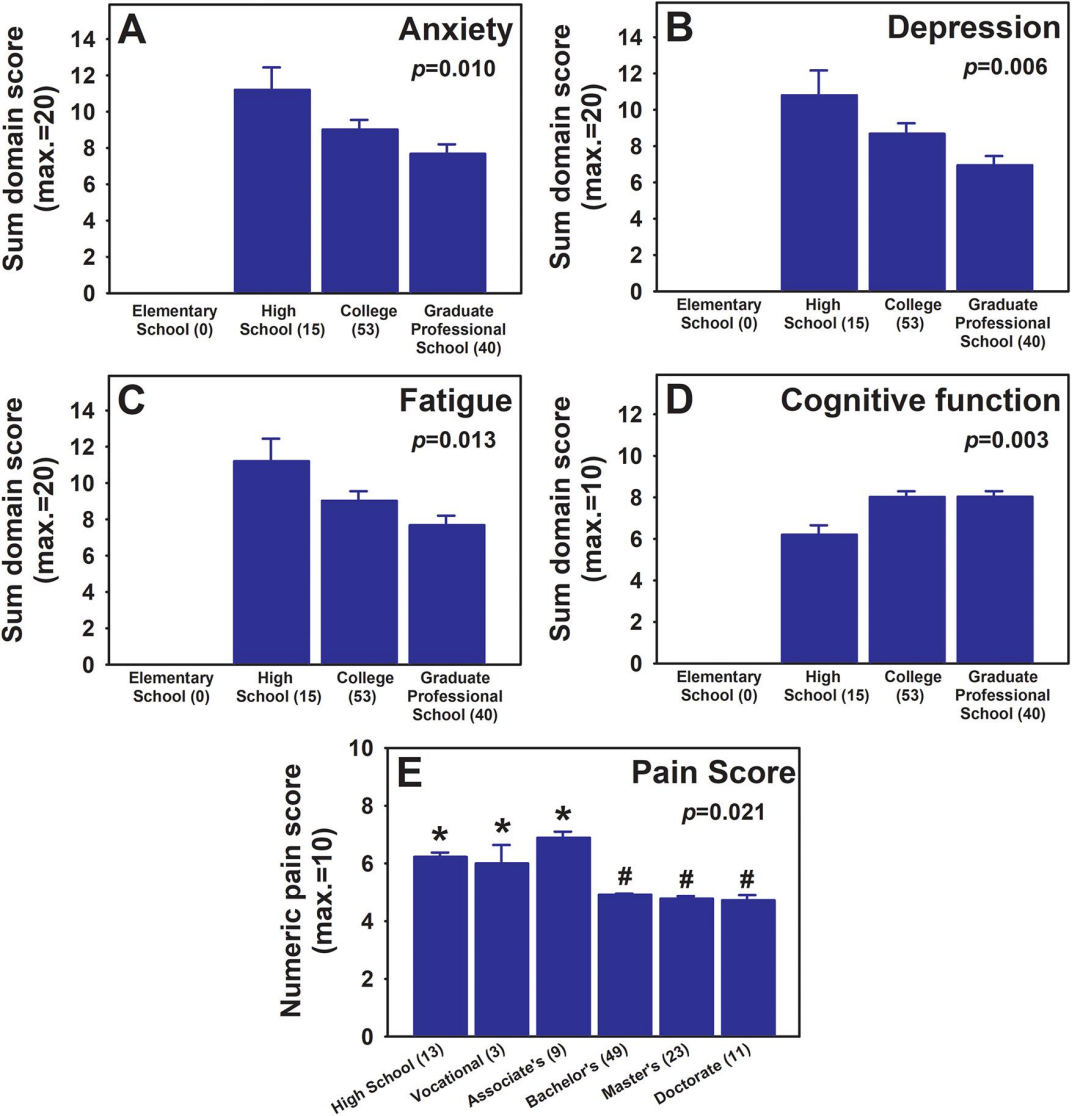
Relationships between education levels and selected PROPr pain outcome measures. PROPr anxiety **(A)**, depression **(B)**, fatigue **(C)**, and cognitive function **(D)** scores across education levels dictated by highest level of school completed are shown. Higher mean scores for anxiety, depression, and fatigue correlate to worse outcomes. The mean sum scores for each indicated outcome domain are presented for each education level (mean ± SEM). The mean numeric pain scores across education levels represented by the highest degree earned are presented in **(E)** (mean ± SEM). For **(A–E)**, one-way ANOVA *p* values are shown in each graph. In **(E)**, *p* < 0.05 between any of the groups indicated by * vs. any groups indicated by #. Numbers of participants within each group are listed with respective group names in parenthesis. Note that higher scores for anxiety, depression, and fatigue correlate to worse outcomes, while higher score for cognitive function correlates to better outcomes. Higher score for numeric pain score correlates to worse pain experienced.

Regarding the education level and pain outcomes, both education level measurements (the highest level of school completed and the highest degree earned) showed significant correlations with PROPr pain outcome domains: anxiety, depression, fatigue, and cognitive function, i.e., higher education levels were associated with better outcomes in all 4 of these measures. Relations between “the highest level of school completed” vs. respective domains are shown in Figure 5 (one-way ANOVA for all; 5A, Anxiety, *p* = 0.010; 5B, Depression, *p* = 0.006; 5C, Fatigue, *p* = 0.013; and Cognitive function, *p* = 0.007). In addition, the highest degree earned was negatively associated with the numeric pain score (Figure 5E, one-way ANOVA, *p* = 0.021) and notably, groups that earned Bachelor's degree and higher showed significantly lower pain scores (Figure 5E, follow up post-hoc test, *p* < 0.001 and *p* < 0.05 between each of the groups with education level below the Bachelor's degree vs. any groups with education level at or above the Bachelor's degree).

In addition, we also examined the relationships between other socio-demographic factors: sex, gender, veteran status, and county of residence (rural vs. urban counties) vs. pain outcome domains. No significant correlations between these factors and any of the PROPr domains for pain outcomes were detected with this initial participant pool.

## 4 Discussion

The long-term goal of our study is to characterize and identify a broader range of SDOH factors that may affect those living with pain in Maine. The state's higher portion of older individuals and its rural geography creates a unique opportunity to study chronic pain considering the higher pain prevalence in individuals with advanced age and geographical barriers to access of health services to manage pain. Furthermore, few studies have explored pain in social context beyond age, sex/gender and race/ethnicity (21). Our analysis aims to not only explore these characteristics but also additional social dimensions as they relate to pain-related outcomes. To achieve this, we established the first pain registry in Maine, PainRegistryforME (now PainRegistryforME2). This report reflects our analysis of the “first-look” of the data. This initial cohort was predominantly white, female, highly educated, and residents of urban counties. Although it has a greater racial diversity than the Maine's general population, it underrepresents the veteran population. All participants met the diagnosis criteria for chronic pain and most participants experienced moderate to severe chronic pain. Our participant pool is overrepresented by individuals at and above 65 and females compared to the census data. This is consistent with many previous reports that chronic pain is more prevalent in older individuals and females (2, 8–11). Regarding pain co-morbidities, besides pain interference and physical functions, fatigue and sleep disturbance are more notable adverse outcomes than depression and anxiety in our participants. This may be related to our community-based data collection approach that analyzed a different respondent population compared to clinic-based settings (22). Further, among our participants, nearly half of them reported low back pain, which is much greater than what was reported for the US adults (28.1%) (23) and the veteran population (10.3%) (24). This may be associated with the type of our participants’ daily activities at home and at work or other demographic characteristics, and requires future investigation. More than a third of individuals believed that their pain originated from previous trauma/injury. This speaks to the importance of injury prevention. This is particularly critical in populations who exhibit risk factors associated with post-traumatic chronic pain, such as those prone to spinal cord injury or disc-vertebra trauma, with history of mental health issues and/or alcohol use disorder, and being female (25). Additionally, our results demonstrated an overall negative impact SDOH risk factors on pain outcomes (Figure 2) in our cohort, which is consistent with previous report (7). Detailed discussion with regard to selected individual SDOH factors are below.

Similar to past research on the impact of pain on different age groups, our analysis showed that older participants with chronic pain demonstrated better mental health outcomes including less anxiety and depression (26). Older adults tend to use a wide variety of coping strategies compared to younger adults including utilizing social support, positive thinking, and pacing themselves such as by taking breaks and moving more slowly in response to their pain (27). A notable finding from our analysis is that older adults with chronic pain demonstrated better cognitive function which conflicts other studies showing an association with a decline in cognitive function specifically related to memory and attention (28). A possible explanation for this could be attributed to the large percentage of individuals with Bachelor's degrees and higher in our current cohort.

Several studies have found racial disparities in the experience and management of chronic pain with Black individuals reporting significantly more disability along with symptoms of depression compared to White individuals (29). There is also evidence that minorities often receive lower quality pain care than non-Hispanic white people (29). Consistent with these reports, our analysis showed that White race was associated with better outcomes in anxiety, depression, and sleep disturbance. A recent study of 600 participants diagnosed with Fibromyalgia syndrome who were predominantly female and 85% White displayed similar racial and sex makeups to our Maine samples. Our observation aligns with the results from this study that found that racial and ethnic minorities experienced greater mood disturbance, depression, and sleep disturbance (30).

Education attainment remains an important component of socioeconomic status (SES). People with lower SES often face barriers to accessing quality medical care as well as adequate social and financial resources, and could not benefit from the schooling process (i.e., time spent before achieving a degree, involving quality of education, interactions with peers and mentors, institutional resources, etc.) to health, thus putting them at risk for poorer health outcomes, and potentially negatively affecting the health of the subsequent generations due to the lack of resources to pursue higher education (31). Although past research has suggested that higher levels of education are correlated with lower pain prevalence (32), few studies have explored beyond prevalence to examine the pain disparities associated with education levels (33). Consistent with the previous study (33), our study also identified lower reported pain scores in individuals with greater levels of educational attainment, with a particular cut-off being with or without a Bachelor's degree. In addition, our analysis found that higher education was associated with better outcomes in depression, anxiety, fatigue, and cognitive function, aligning with a previous study of patients with fibromyalgia (34). Factors that may explain our specific findings include that higher education level allows for richer resources and better medical care of their pain which potentially translates into lower fatigue levels and improved mental health. Furthermore, having access to social and economic resources could result in fewer concerns about pain and depressive symptoms, both of which have been linked to the lower pain levels in patients with fibromyalgia (34). Better cognitive function in those with higher education in our sample may reflect the direct benefits of education as well as the indirect effects of improved overall health (31).

Although no significant correlations were found between the following factors and any of the eight pain-related outcome domains, it is important to acknowledge why these characteristics were chosen to explore based on existing studies on chronic pain. Multiple studies have suggested that women are more likely to experience a variety of chronic pain syndromes at higher intensities than men (35). Our results showed that most participants identified as female which is consistent with CDC national data on chronic pain (2) and our previous report on Maine's population (8). Sex refers to the biological attributes including genetics and hormones while gender refers to more of a social role. Hormones such as estrogen play a role in signaling pathways and anatomical differences may account for differences in pain perception. With gender, it is suggested that because of societal norms, women are more inclined to report pain and have more maladaptive coping mechanisms (36). Veteran status was a demographic factor that was of interest because military veterans are more likely to have chronic pain compared to the general population (37) and Maine has a significant veteran population (7.2% according to the census data). Chronic pain in this population becomes complicated by mental health conditions and substance use disorders which are common comorbidities (38). However, our results only included 6 (5.5%) veterans, which is an underrepresentation based on Maine's census and provides insufficient power for robust analysis. Pain prevalence is higher in rural areas compared to urban areas (39). Rural areas often face limited economic opportunities, which can restrict access to healthcare services and health-enhancing activities (40). Although Maine is largely rural, making this demographic relevant, most of our participants resided in urban counties that reduced the statistical power of our analysis significantly.

Our findings should be interpreted with several limitations. The pain registry was created at the University of New England and widely distributed on our two campuses in Biddeford and Portland and surrounding mostly urban areas, accounting for the most densely populated region of the state. This may account for skewed data comparing to Maine's general population particularly pertaining to more participants with younger age, urban residence, non-white ethnicity, and higher education levels, with fewer participants being veterans and living in rural counties. Despite using a multi-modal recruitment strategy, enrollment was dependent on voluntary response and how active dissemination channels were in promoting the study. Efforts were made to distribute the flyers to collaborating sites but it is uncertain whether those on the receiving end distributed the flyers and whether the recruitment efforts were sustained over time. Current participant profiles call for further recruitment of Mainers living with greater SDOH risk factors, particularly those living in rural counties. Additional outreach and recruitment plans are being designed to capture individuals with these characteristics. With the continued effort, we believe that our participant pool will evolve and reflect the general population in Maine over time. The study utilizes data collected through an online survey which is subjected to self-selection and self-reporting biases. Participant burden related to the length of the survey and its online platform may have influenced who chose to participate. Overall, the survey usually took the participants 10–30 min to complete. As we provided an option “Prefer not to answer” for each question, the survey completion rate for each participant is relatively high. Another challenge with an online survey is the risk of responses from automated sources which led to temporary interruptions in data collection for this registry while measures were taken to ensure data integrity. Although the registry is designed to be longitudinal, this current report performed a cross-sectional data analysis and does not allow for further causal inference. Interpretation of these results should be undertaken with caution as this analysis is done on preliminary “first look” data in an ongoing and active registry. Demographic and SDOH factors were selected based on previously known literature and research on chronic pain. With continued data collection and larger sample sizes, the patterns observed within this registry cohort are subject to change and may evolve or strengthen. Therefore, comparisons with our dataset to state and national estimates are presented to contextualize the current findings. Further, other factors collected in the registry, such as those related to pain management and other SDOH factors (such as health insurance, housing conditions, food intake, etc.) may help to identify additional impact of SDOH on chronic pain and mediators between selected SDOH factors and chronic pain.

In summary, although the initial cohort from the pain registry identified several relevant SDOH factors in pain, continued data collection from PainRegistryforME is necessary to understand the pain population in Maine and how specific demographic and SDOH characteristics could influence individuals’ chronic pain experiences. The higher prevalence of pain and related comorbidities reported by certain social groups can inform clinicians when assessing patient's risks for developing chronic pain and identifying appropriate preventative and treatment options. Further results can guide public health strategies and interventions for pain prevention and management in Maine.

## Data Availability

The raw data supporting the conclusions of this article will be made available by the authors, without undue reservation.

## References

[1] Gaskin DJ Richard P. The economic costs of pain in the United States. J Pain. (2012) 13(8):715–24. 10.1016/j.jpain.2012.03.009 22607834

[2] Lucas JW Sohi I. Chronic pain and high-impact chronic pain in U.S. adults, 2023. NCHS data Brief. (2024) (518):CS355235. 10.15620/cdc/169630 39751180 PMC11726267

[3] De La Rosa JS Brady BR Herder KE Wallace JS Ibrahim MM Allen AM The unmet mental health needs of U.S. adults living with chronic pain. Pain. (2024) 165(12):2877–87. 10.1097/j.pain.0000000000003340 39073375 PMC11562766

[4] Mills SEE Nicolson KP Smith BH. Chronic pain: a review of its epidemiology and associated factors in population-based studies. Br J Anaesth. (2019) 123(2):e273–e83. 10.1016/j.bja.2019.03.023 31079836 PMC6676152

[5] Karran EL Fryer CE Middleton JW Moseley GL. Exploring the social determinants of health outcomes for adults with low back pain or spinal cord injury and persistent pain: a mixed methods study. J Pain. (2022) 23(9):1461–79. 10.1016/j.jpain.2022.04.001 35429673

[6] Kapos FP Craig KD Anderson SR Bernardes SF Hirsh AT Karos K Social determinants and consequences of pain: toward multilevel, intersectional, and life course perspectives. J Pain. (2024) 25(10):104608. 10.1016/j.jpain.2024.104608 38897311 PMC11402600

[7] Karran EL Grant AR Moseley GL. Low back pain and the social determinants of health: a systematic review and narrative synthesis. Pain. (2020) 161(11):2476–93. 10.1097/j.pain.0000000000001944 32910100

[8] Malon J Shah P Koh WY Cattabriga G Li E Cao L. Characterizing the demographics of chronic pain patients in the state of Maine using the Maine all payer claims database. BMC public Health. (2018) 18(1):810. 10.1186/s12889-018-5673-5 29954350 PMC6022454

[9] Dahlhamer J Lucas J Zelaya C Nahin R Mackey S DeBar L Prevalence of chronic pain and high-impact chronic pain among adults - United States, 2016. MMWR Morb Mortal Wkly Rep. (2018) 67(36):1001–6. 10.15585/mmwr.mm6736a2 30212442 PMC6146950

[10] Johannes CB Le TK Zhou X Johnston JA Dworkin RH. The prevalence of chronic pain in United States adults: results of an internet-based survey. J Pain. (2010) 11(11):1230–9. 10.1016/j.jpain.2010.07.002 20797916

[11] Zajacova A Grol-Prokopczyk H Zimmer Z. Pain trends among American adults, 2002-2018: patterns, disparities, and correlates. Demography. (2021) 58(2):711–38. 10.1215/00703370-8977691 33834222 PMC8035485

[12] Harris PA Taylor R Minor BL Elliott V Fernandez M O'Neal L The REDCap consortium: building an international community of software platform partners. J Biomed Inform. (2019) 95:103208. 10.1016/j.jbi.2019.103208 31078660 PMC7254481

[13] Harris PA Taylor R Thielke R Payne J Gonzalez N Conde JG. Research electronic data capture (REDCap)–a metadata-driven methodology and workflow process for providing translational research informatics support. J Biomed Inform. (2009) 42(2):377–81. 10.1016/j.jbi.2008.08.010 18929686 PMC2700030

[14] LaForge K Gold R Cottrell E Bunce AE Proser M Hollombe C How 6 organizations developed tools and processes for social determinants of health screening in primary care: an overview. J Ambul Care Manage. (2018) 41(1):2–14. 10.1097/JAC.0000000000000221 28990990 PMC5705433

[15] Manchanda R Gottlieb L. Upstream Risks Screening Tool and Guide V2.6. Los Angeles, CA: HealthBegins (2015). [This work is licensed under Creative Commons Attribution-NonCommercial-ShareAlike 4.0 International License]. Available online at: https://www.medchi.org/Portals/18/Files/Practice%20Services/SDoH%20Screening%20Tool.pdf?ver=2023-08-10-131750-180 (Accessed 2015).

[16] HealthMeasures. Intro to PROMIS, List of Adult Measures. https://www.healthmeasures.net/: Evanston, IL: Northwestern University (2022). [Funding for HealthMeasures was provided by the National Institutes of Health grant U2C CA186878]. Available online at: https://www.healthmeasures.net/explore-measurement-systems/promis/intro-to-promis/list-of-adult-measures (Accessed April 27, 2022).

[17] Dewitt B Feeny D Fischhoff B Cella D Hays RD Hess R Estimation of a preference-based summary score for the patient-reported outcomes measurement information system: the PROMIS(®)-preference (PROPr) scoring system. Med Decis Making. (2018) 38(6):683–98. 10.1177/0272989X18776637 29944456 PMC6502464

[18] USDA_Economic_Research_Service. 2024 Urban Influence Codes. ers.usda.gov: Washington, DC: USDA Economic Research Service (2024). Available online at: https://www.ers.usda.gov/data-products/urban-influence-codes (Accessed January 7, 2025; November 25, 2025).

[19] LuminaFoundation. 2023 Maine Report. https://strongernation.luminafoundation.org/credentials-of-value: Lumina Foundation (2023). Available online at: https://www.maine.gov/swb/docs/2023/meetings/06092023/MaineSpark-2023Report.pdf (Accessed November 25, 2025).

[20] Treede RD Rief W Barke A Aziz Q Bennett MI Benoliel R Chronic pain as a symptom or a disease: the IASP classification of chronic pain for the international classification of diseases (ICD-11). Pain. (2019) 160(1):19–27. 10.1097/j.pain.0000000000001384 30586067

[21] Grol-Prokopczyk H Huang R Yu C Chen YA Kaur S Limani M Over 50 years of research on social disparities in pain and pain treatment: a scoping review of reviews. Pain. (2025) 166(11):2458–72. 10.1097/j.pain.0000000000003676 40553634 PMC12353892

[22] Horn ME Reinke EK Yan X Luo S Bolognesi M Reeve BB Use of patient-reported outcomes measurement information system (PROMIS) measures to characterise health status for patients seeking care from an orthopaedic provider: a retrospective cohort study. BMJ open. (2021) 11(9):e047156. 10.1136/bmjopen-2020-047156 34475157 PMC8413970

[23] Pal S. Trends in pain prevalence. US Pharm. (2016) 41(3):20.

[24] Taylor KA Kapos FP Sharpe JA Kosinski AS Rhon DI Goode AP. Seventeen-year national pain prevalence trends among U.S. Military veterans. J Pain. (2024) 25(5):104420. 10.1016/j.jpain.2023.11.003 37952861 PMC11184511

[25] Daoust R Paquet J Moore L Émond M Gosselin S Lavigne G Early factors associated with the development of chronic pain in trauma patients. Pain Res Manag. (2018) 2018:7203218. 10.1155/2018/7203218 29666666 PMC5830982

[26] You DS Ziadni MS Hettie G Darnall BD Cook KF Korff V Comparing perceived pain impact between younger and older adults with high impact chronic pain: a cross-sectional qualitative and quantitative survey. Front Pain Res (Lausanne). (2022) 3:850713. 10.3389/fpain.2022.850713 35465295 PMC9030949

[27] Molton I Jensen MP Ehde DM Carter GT Kraft G Cardemas DD. Coping with chronic pain among younger, middle-aged, and older adults living with neurological injury and disease. J Aging Health. (2008) 20(8):972–96. 10.1177/0898264308324680 18791184 PMC2716650

[28] van der Leeuw G Eggermont LH Shi L Milberg WP Gross AL Hausdorff JM Pain and cognitive function among older adults living in the community. J Gerontol A Biol Sci Med Sci. (2016) 71(3):398–405. 10.1093/gerona/glv166 26433218 PMC5013972

[29] Anderson KO Green CR Payne R. Racial and ethnic disparities in pain: causes and consequences of unequal care. J Pain. (2009) 10(12):1187–204. 10.1016/j.jpain.2009.10.002 19944378

[30] Marr NC Van Liew C Carovich TF Cecchini GA McKinley LE Cronan TA. The effects of racial/ethnic minority Status on sleep, mood disturbance, and depression in people with fibromyalgia. Psychol Res Behav Manag. (2020) 13:343–53. 10.2147/PRBM.S242699 32368163 PMC7174195

[31] Zajacova A Lawrence EM. The relationship between education and health: reducing disparities through a contextual approach. Annu Rev Public Health. (2018) 39:273–89. 10.1146/annurev-publhealth-031816-044628 29328865 PMC5880718

[32] Grol-Prokopczyk H. Sociodemographic disparities in chronic pain, based on 12-year longitudinal data. Pain. (2017) 158(2):313–22. 10.1097/j.pain.0000000000000762 28092650 PMC5242384

[33] Zajacova A Rogers RG Grodsky E Grol-Prokopczyk H. The relationship between education and pain among adults aged 30–49 in the United States. J Pain. (2020) 21(11-12):1270–80. 10.1016/j.jpain.2020.03.005 32574784 PMC7722114

[34] Fentazi D Pester BD Yamin JB Jamison RN Edwards RR Meints SM. Why is low educational attainment linked to worse pain and function in fibromyalgia? J Pain. (2025) 27:104764. 10.1016/j.jpain.2024.104764 39725050 PMC11807746

[35] Racine M Tousignant-Laflamme Y Kloda LA Dion D Dupuis G Choinière M. A systematic literature review of 10 years of research on sex/gender and experimental pain perception - part 1: are there really differences between women and men? Pain. (2012) 153(3):602–18. 10.1016/j.pain.2011.11.025 22192712

[36] Templeton KJ. Sex and gender issues in pain management. J Bone Joint Surg Am. (2020) 102 Suppl 1:32–5. 10.2106/JBJS.20.00237 32251123

[37] CDC. Quickstats: percentage of adults aged ≥20 years who had chronic pain, by veteran status and age group - national health interview survey, United States, 2019. MMWR Morb Mortal Wkly Rep. (2020) 69(47):1797. 10.15585/mmwr.mm6947a6 33237896 PMC7727604

[38] Higgins DM Kerns RD Brandt CA Haskell SG Bathulapalli H Gilliam W Persistent pain and comorbidity among operation enduring freedom/operation Iraqi freedom/operation new Dawn veterans. Pain Med. (2014) 15(5):782–90. 10.1111/pme.12388 24548466

[39] Yang Y Sun F Zimmer Z Zajacova A Huang R Grol-Prokopczyk H Chronic pain prevalence and trends in urban, suburban, and rural areas among American adults aged 55+, 1998–2022. Soc Sci Med. (2025) 385:118625. 10.1016/j.socscimed.2025.118625 41027294 PMC12588185

[40] Johnston KJ Wen H Maddox J E K. Lack of access to specialists associated with mortality and preventable hospitalizations of rural medicare beneficiaries. Health Aff (Millwood). (2019) 38(12):1993–2002. 10.1377/hlthaff.2019.00838 31794307

